# From viewership to appetite: a study on Mukbang watching prevalence and its influence on hedonic hunger among university nursing students

**DOI:** 10.1186/s12912-025-03287-3

**Published:** 2025-06-17

**Authors:** Amel Attia Abd Elghaffar Moustafa, Mariam Roshdy Elkhayat, Abeer Abd El-Aziz Madian, Ahmed Abdellah Othman, Mahmoud Abdelwahab Khedr, Asmaa Hamed Mohamed

**Affiliations:** 1 Community Health Nursing Department, Faculty of Nursing, Damanhour University, Damanhour, Egypt; 2 Public Health and Community Medicine, Faculty of Medicine, Assiut University, Assiut, Egypt; 3 Nursing Administration Faculty of Nursing -Sohag University, Sohag, Egypt; 4 Psychiatric and Mental Health Nursing, Alexandria University, Alexandria, Egypt

**Keywords:** Mukbang, Hedonic hunger, University nursing students

## Abstract

**Background:**

Mukbang, a popular online activity from South Korea, involves hosts consuming large quantities of food while engaging with viewers. Concerns about its potential impact on eating behaviors have been raised, particularly among vulnerable populations such as university nursing students.

**Aim:**

This study investigates the prevalence of Mukbang watching and its influence on hedonic hunger, characterized by cravings for pleasurable foods independent of physiological hunger.

**Methods:**

A cross-sectional study was conducted among 746 undergraduate nursing students at Damanhour University, Egypt. Data were collected through an online survey that assessed socio-demographic information, Mukbang watching habits, and hedonic hunger using the Mukbang Addiction Scale and the Power of Food Scale. Data were collected from December 2024 to February 2025.

**Results:**

The mean age of participants was 21.04 ± 1.55 years, with 68.4% being female. A significant percentage (68.5%) reported watching food-related videos, spending an average of 13.61 ± 42.46 min daily on such content. The mean score for Mukbang addiction was 11.02 ± 4.91, while the mean hedonic hunger score was 39.79 ± 13.41. A positive correlation was found between Mukbang addiction and hedonic hunger (*r* = 0.136, *P* < 0.001). Multiple regression analysis revealed that daily internet usage (B = 7.85, *P* < 0.001) and Mukbang addiction (B = 0.35, *P* = 0.007) significantly predicted hedonic hunger.

**Conclusion:**

The study highlights the prevalence of Mukbang watching among nursing students and its significant association with increased hedonic hunger. These findings underscore the need for targeted interventions to promote healthy eating habits, particularly in rising food-related media consumption among young adults. Understanding the influence of such media is essential for addressing potential health risks linked to unhealthy eating behaviors.

**Clinical trial number:**

Not applicable.

## Background

Currently, numerous online activities are emerging, one of which is watching individuals eat, known as Mukbang. This term originates from South Korea, combining “meokneun” (eating) and “bang song” (broadcast). Mukbang first appeared online in 2008 on a South Korean TV channel and expanded to a global audience in 2015 [1]. Mukbang is a live or prerecorded program in which the host engages with the audience while consuming a large quantity of food [2, 3]. Detractors contend that Mukbang can cause overeating and harm viewers by increasing food consumption through imitation or social comparison, changing viewers’ perceptions of eating habits by modeling unhealthy behaviors [4]. Despite its growing popularity, concerns have been raised about the potential impact of Mukbang watching on eating behaviors and food choices [5].

During early adulthood, students often display unhealthy eating habits, as this is a time when they gain more independence in their dietary choices. This can lead to poor nutrition, as they frequently eat out, have irregular meal patterns, operate on limited budgets, lack proper nutritional knowledge, and consume a significant amount of fast food [6]. Additionally, in Egypt, 90% of university students were reported to use smartphones in 2022 [7]. As a result, students may be particularly vulnerable to the negative effects of watching Mukbang, given the rapidly increasing availability of such content on mobile devices [8].

Research has shown that exposure to food-related media can increase cravings and appetite, chiefly for high-calorie and high-fat foods [9, 10]. Moreover, a study has found that individuals who engage in emotional eating, such as those who experience stress or anxiety, may be more susceptible to the influence of food-related media. Nursing university students, in particular, may be vulnerable to the effects of Mukbang watching due to their demanding academic schedules, high stress levels, and limited time for self-care [11].

Hedonic hunger (HH) is the term used to describe an imbalance of hormones that regulate eating that causes a persistent urge to eat enjoyable foods—foods that are heavy in fat, sugar, and salt—even when there is no physiological hunger [12]. The level of HH varies among individuals, and those who score the highest on the HH scale may have problematic behavioral and physiological characteristics. True homeostatic hunger only occurs a few times daily, primarily concerning the energy balance regulation of peripheral hormones (leptin and ghrelin) [13]. It has been demonstrated that those with greater HH tend to lose control when eating, which may lead to the development of binge eating disorder, which, if untreated, may eventually result in obesity [14].

The mechanisms underlying the effect of Mukbang watching on hedonic hunger are not fully understood, but several factors may contribute to this phenomenon. First, mukbang videos often feature large portions of high-calorie, high-fat, and high-sugar foods, activating the brain’s reward centers and increasing cravings [11]. Second, Mukbang’s social interaction and communal eating aspect may enhance the enjoyment and pleasure of eating, leading to increased hedonic hunger [15]. Finally, the repetitive and ritualistic nature of mukbang watching may contribute to the development of conditioned responses to food cues, increasing hedonic hunger and food cravings [16].

However, it is essential to investigate this phenomenon, particularly among populations vulnerable to Mukbang’s effects, such as nursing university students. Moreover, limited research focuses explicitly on the university nursing student population and the impact of Mukbang watching on their eating behaviors. So, this study aims to fill this gap by evaluating the prevalence of mukbang watching among university nursing students and examining how it influences their hedonic hunger. Understanding these factors can provide valuable insights into the eating behaviors of future healthcare professionals and inform interventions to promote healthier eating habits.

## Significance of study

This study provides valuable insights into this popular online activity’s potential risks and consequences. Also, The World Health Organization (2020) [17] has emphasized the need for effective strategies to promote healthy eating habits and prevent diet-related diseases. By understanding the impact of Mukbang watching on hedonic hunger, healthcare professionals and nutrition educators can develop targeted interventions to promote healthy eating habits and reduce the risk of overeating and weight gain [18].

Moreover, this study highlights the importance of considering eating behaviors’ social and cultural context. Mukbang watching is a popular online activity involving social interaction and communal eating, enhancing the enjoyment and pleasure of eating. So, this study provides insights into the complex interplay between social, cultural, and psychological factors that influence eating behaviors [15].

Finally, this study has implications for developing effective treatments for eating disorders. Hedonic hunger is a key feature of several eating disorders, including binge eating disorder and bulimia nervosa. By understanding the impact of Mukbang watching on hedonic hunger, healthcare professionals can develop targeted interventions to reduce the risk of overeating and promote healthy eating habits [12].

### Methods

#### Aim of the study

The primary objective is to evaluate the impact of Mukbang viewing on hedonic hunger (i.e., the craving for food as a source of pleasure rather than for nutritional needs) within this population.

This study aims to investigate the prevalence of Mukbang watching among university nursing students and determine its potential influence on hedonic hunger. Specifically, the research seeks to assess how frequently nursing students engage in Mukbang viewing, examine their perceptions of food-related stimuli, and understand if there is a link between watching Mukbang and an increase in emotional or pleasure-driven eating (hedonic hunger).

#### Research questions

1. What is the prevalence of Mukbang watching among university nursing students?
2. How does Mukbang watching influence hedonic hunger in university nursing students?
3. Is there a significant relationship between mukbang watching and hedonic hunger among university nursing students?
4. What key factors predict hedonic hunger among nursing students, as determined through linear regression analysis?

#### Study design

This study employed a cross-sectional design to assess the prevalence of Mukbang watching among nursing students in the Faculty of Nursing, Damanhour University in El-Beheira Governorate, Egypt. A cross-sectional study is ideal for this research because it enables data collection at a single point, providing a snapshot of the prevalence of Mukbang consumption and its association with hedonic hunger in this group.

#### Study population

The study population consisted of 746 undergraduate nursing students enrolled in the aforementioned setting. The study aimed for a representative sample to ensure diversity in responses, including students from different years of study, both male and female participants. Students must be 18 years or older to participate. Students with diagnosed eating disorders, metabolic disorders, or other medical conditions that could confound the results were excluded from the study.

#### Sample size

The researchers utilized Open Epi, Version 3, an open-source calculator, to calculate the necessary sample size for the study. The total number of nursing students at the universities surveyed was 6,850. The calculation parameters included a hypothesized frequency of the outcome factor (p) set at 40%, a margin of error of ± 4%, a confidence level of 98%, and a design effect (DEFF) of 1, which is appropriate for cluster surveys. The sample size was calculated using the formula:

Sample size (n)=[DEFF * N * p * (1-p)] / [(d^2 / Z^2) * (N-1) + p * (1-p)]. As a result, the required sample size for this study was found to be 746.

#### Study tools

The following three instruments were used to get the required data.

##### Tool I

The study tool designed to collect socio-demographic data incorporates several key variables that comprehensively understand participants’ backgrounds and behaviors. These include basic demographic information such as age, sex, marital status, and residence. Additionally, participants are asked about weight and height, which contribute to an analysis of their body mass index (BMI). Economic factors are assessed through questions on their income, while digital engagement is measured by inquiries into daily internet usage and the devices used. The tool also assesses the influence of food-related digital content by asking participants whether they watch Muckbang videos and how much time they spend on such content daily.

##### Tool II Mukbang Addiction Scale (MAS)

The Mukbang Addiction Scale (MAS), consisting of six items [4], was employed to evaluate mukbang addiction (e.g., “In the past year, how often have you felt an increasing urge to watch mukbang?“). The MAS was created based on the components model of addiction, a widely recognized framework in the field of behavioral addictions [19]. Each item was rated on a 5-point Likert scale (1 = very rarely, 5 = very often), with higher scores reflecting a greater risk of mukbang addiction. In this study, no arbitrary cut-off points were used; instead, the interpretation was based on the natural distribution of the total scores. This method is congruent with normal standards for Likert-based behavioral measures, in which higher cumulative values indicate a greater risk of the targeted behavior. The scale demonstrated good reliability, with a Cronbach’s alpha (α) of 0.87.

##### Tool III: The power of food scale (PFS)

The Power of Food Scale (PFS) was utilized to evaluate participants’ hedonic motivation for food [20]. The PFS assesses the attraction to highly palatable foods in situations where individuals are not physiologically hungry, a concept referred to as hedonic hunger. It gauges the frequency of a strong desire to consume such foods rather than the actual quantity or frequency of consumption.

The scale comprises 15 items that evaluate appetite and motivation to eat palatable foods across three domains: food available (e.g., “I often think about food even when I’m not physically hungry”); food present (e.g., “When I see or smell a food I like, I have a strong urge to eat it”); and food tasted (e.g., “Before tasting a favorite food, I feel intense anticipation”). This construct is believed to contribute to excessive food intake beyond metabolic needs.

Responses are rated on a 5-point Likert scale, ranging from 1 (do not agree at all) to 5 (strongly agree). The overall mean score from the questionnaire indicates how responsive participants are to their food environment, with possible scores ranging from 15 to 75. The PFS is scored by summing all items and dividing by the number of items. Higher scores are associated with participants’ hedonic motivation to eat palatable food. Rather than adopting predetermined cut-offs, we interpreted results based on distribution, as is generally accepted in Likert-scale assessments. It has demonstrated reliability, with a Cronbach’s alpha (α) of 0.89 [21].

### Pilot study

A pilot study with 10% of the participants (75 nursing students) was conducted before the primary research to evaluate the tools’ practicality and applicability and make any necessary adjustments. The study sample did not include any students participating in the pilot phase.

### Data collection

An online survey served as the primary data gathering for the research study. Questions that assessed the frequency of Mukbang watching among participants and its possible influence on the experience of hedonic hunger were incorporated into a structured online form. The survey comprised sections addressing demographic information, patterns of Mukbang viewing, and the subjective experiences related to hedonic hunger. To guarantee accessibility, simplicity of participation, and effective data collection, the online format was selected, enabling respondents to submit their information at their convenience and anonymously. The survey was disseminated using online platforms that university nursing students frequently utilize, such as social media groups and email, guaranteeing broad participation. By reducing the logistical limitations of conventional, in-person surveys, this approach made it easier to gather thorough and trustworthy data. Data were collected from December 2024 to February 2025.

### Ethical issues

The research ethics committee of Damanhour University’s nursing faculty provided ethical permission (N:108-D/ 12/ 2024). The study rigorously followed the ethical guidelines outlined in the Helsinki Declaration to preserve the integrity of the research. The Participants Information Sheets (PIS) were located on the first page of the online survey and included information on the study’s objectives, voluntary nature, potential hazards, and expected benefits. Participation in this anonymous online survey was entirely voluntary. Before beginning the online survey, participants must read the first two parts and check the permission form box.

### Data analysis

SPSS 26.0 (IBM Inc., Chicago, IL, USA) was used for data analysis to evaluate the survey responses from the 746 nursing students. The participants’ general characteristics and the scores were obtained on various scales. Descriptive statistics were assessed frequencies (No/%) and mean ± standard deviations (S.D.), which were utilized to summarize. The correlations between Mukbang Watching addiction and hedonic hunger were examined by Pearson’s correlation analysis utilized to assess. A linear regression analysis test was used to test the predictor factor for hedonic hunger.

## Results

Table 1 shows that the mean score of the studied participants’ age was 21.039 **±** 1.549, 68.4% were female, and the mean score of participants’ weight and height were 68.956 **±** 14.8394 and 166.316 **±** 166.316 respectively. Also, 92.2% of them were single, 71.3% were from rural areas, 75.2% hadn’t special income, 48.3% were satisfied with my weight, 45.8% used the internet about 4–5 h or more daily, 84.6% used to connect with the Internet through telephone, 68.5% of them watch food videos and review videos about food and restaurants and the mean score of minutes a day watch food videos was 13.609 **±** 42.463.

**Table 1:** Personal data (*n* = 746)

Personal data	Categories	No	%
Age	Mean **±** SD	21.039 **±** 1.549	
Gender	Male	236	31.6
	female	510	68.4
weight	Mean **±** SD	68.956 **±** 14.8394	
height	Mean **±** SD	166.316 **±** 166.316	
Marital status	single	688	92.2
	married	58	7.8
Residence	Rural	532	71.3
	Urban	214	28.7
Have you had special Income	Yes	185	24.8
	No	561	75.2
Weight status	I want to lose weight	22	29.5
	I want to increase weight	166	22.3
	I’m satisfied with my weight	360	48.3
How long do you use the Internet daily?	1 h and less	63	8.4
	2–3	341	45.7
	4–5 h and more	342	45.8
What device is used to use the Internet?	Computer	5	0.7
	Telephone	631	84.6
	Computer and telephone	110	14.7
Do you watch food videos and review videos about food and restaurants?	Yes	511	68.5
	no	235	31.5
How many minutes a day do you watch food videos?	Mean **±** SD	13.609 **±** 42.463	

Table 2 reveals that the mean score of the Mukbang watching addiction among the studied participants was 11.0241 **±** 4.91014, and the mean score for hedonic hunger among the studied participants was 39.7882 **±** 13.41483.

**Table 2:** Descriptive analysis of the study variables (*n* = 746)

Study variables	*N*. items	Minimum	Maximum	Mean	SD
Mukbang Watching addiction	6	6.00	30.00	11.0241	4.91014
Hedonic Hunger	15	15.00	75.00	39.7882	13.41483

SD = Standard Deviation

Table 3: Illustrates that there was a statistically significant positive correlation between participants Mukbang watching addiction and hedonic hunger at (*r* = 0.136^**^, *P* < 0.001).

**Table 3:** Correlation analysis of the study variables (*n* = 746)

Study variables		Mukbang watching	Hedonic hunger
Mukbang watching addiction	r	1	0.136^**^
	P		< 0.001
Hedonic Hunger	r	0.136^**^	1
	P	< 0.001	

**r = Pearson Correlation**, ** Correlation is highly significant at the 0.01 level (2-tailed)

Table 4: Clarifies the predictor factors for Hedonic Hunger; from table results many factors have direct statistical significant effect on Hedonic Hunger as special income at (B = 3.809, t = 3.569, *P* < 0.001) used the internet daily at (B = 7.854, t = 11.289, *P* < 0.001), watch food videos and review videos at (B = 12.674, t = 9.711, *P* < 0.001), special income at (B = 0.345, t = 2.718, *P* = 0.007).

**Table 4:** Liner regression analysis for predicting factors for hedonic hunger among nursing students (*n* = 746)

Model	Unstandardized coefficients		Standardized coefficients	t	Sig.	95.0% confidence interval for B	
	B	Std. error	Beta			Lower bound	Upper bound
(Constant)	88.059	11.798		7.464	0.000	65.941	111.714
age	− 0.354-	0.293	− 0.041-	-1.209-	0.227	− 0.931-	0.218
Gender	-1.271-	1.169	− 0.044-	-1.087-	0.277	-3.577-	1.010
weight	0.051	0.033	0.056	1.535	0.125	− 0.014-	0.117
height	− 0.061-	0.041	− 0.058-	-1.495-	0.135	− 0.143-	0.018
Marital status	1.028	1.654	0.021	0.622	0.534	-2.230-	4.260
Residence	0.545	0.967	0.018	0.564	0.573	-1.357-	2.436
Special Income	3.809	1.067	0.123	3.569	< 0.001	1.713	5.902
Weight status	0.399	0.539	0.026	0.740	0.459	0.667	1.444
use the Internet daily	7.854	0.696	0.370	11.289	< 0.001	9.242	6.523
device is used to use the Internet	-1.381-	1.269	− 0.038-	-1.088-	0.277	3.883	1.097
watch food videos and review videos	12.674	1.305	0.437	9.711	< 0.001	15.230	10.109
How many minutes a day do you watch food videos	0.013	0.011	0.042	1.270	0.204	0.007	0.034
Mukbang Watching addiction	0.345	0.126	0.126	2.718	0.007	0.593	0.097

*R* = 0.518, R Square = 0.269, F = 20.419, *P* < 0.001

## Discussion

Nowadays, college students use social media widely, influencing and shaping their behaviors across various aspects of life, including their eating manners [22]. Thus, the current study aimed to go in-depth to understand the impact of Mukbang watching addiction on hedonic hunger among young adults.

The age of the included students was 21.03 ± 1.54years, with a majority being female (68.4%) and single (92.2%), with a significant proportion from rural areas (71.3%), and 75.2% reported to have no special income, which could limit their ability to purchase healthier food options, potentially exacerbating issues related to hedonic hunger and overeating [23]. These demographic factors are crucial for understanding the context of the study population. Additionally, this criteria was consistent with previous studies investigating internet usage and eating behaviors among young adults [24].

The current study verified the extensive internet use among participants, as 45.8% recorded 4–5 h or more using the internet daily, principally through their phones (84.6%). This high level of internet usage aligns with other publications [25, 26]. Additionally, 68.5% of participants watched food videos and review videos about food and restaurants, with an average of 13.6 ± 42.4 min per day spent watching these videos. This behavior is indicative of the growing popularity of Mukbang and food-related content on social media platforms, which attract a lot of young people [27]; this could stimulate their hunger sensation without their need to have meals this time, this was proved by the presence of positive correlation between Mukbang watching addiction and hedonic hunger (*r* = 0.136, *P* < 0.001) and multivariate linear regression (B = 0.345, *P* = 0.007)., This implies and confirms that individuals who regularly watch mukbang videos may experience heightened hedonic hunger fueled by the enjoyment and satisfaction gained from observing others eat. This is supported by prior research indicating that mukbang viewing can influence food preferences and eating behaviors [24], keeping them riskier to consume fast food, sweetened beverages, and highly caffeinated drinks, especially young adolescents affected by influencers, emphasizing the importance of addressing emerging cultural trends and developing relevant health policies [28].

The current study emphasized the interaction between various socio-demographic variables of Egyptian students (as income, marital status, and rural residence), social media habits (internet and food video exposure up to Mukbang video addiction) with nutritional psychological responses (hedonic hunger), by using multivariable regression analysis, which showed that frequent internet use, watching food videos, having an income, and mukbang video addiction were all significant predictors of hedonism more enormous among Egyptian university students. This reinforces previous studies [4, 28, 29] that affirm that mukbang viewing enhances food cravings and eating motivation through sensory stimulation [30]. Additionally, it established that **hedonic eating behavior is not in isolation but within a matrix of digital exposure and socio-economic determinant**s as included Egyptian students were mostly female, single, and from rural areas, with no special income, highlighting their **psychosocial vulnerability** and economic strains, that may affect their ability to had healthier food options. This restriction could aggravate the effects of hedonic hunger, directing them towards more accessible, calorie-dense foods, which present more in Egypt market [31].

The current results suggest that digital media exposure, especially food shows and socio-economic status, could play a vital role in stimulating hedonic eating. This warrants further research into the psychological and neurobiological mechanisms underlying these associations with different outcomes.

One of the critical limitations of this study is that the use of a cross-sectional design precludes the determination of causal relationships and the temporal sequencing of variables. Consequently, further research employing longitudinal designs is necessary to validate the results presented in this study,

Despite these limitations, there are many strengths: first, to our knowledge, it is the first study done in Egypt. Second, the study had a good representative sample, providing insights into vulnerable, exposed young university adolescents. Third, by employing validated psychometric tools linking Mukbang watching addiction and hedonic hunger among young adults, evidenced that **Mukbang video addiction** is a significant **predictor of hedonic hunger**, independent of other factors such as income, time spent online, and frequency of watching food-related content, by Providing significant **positive correlation and multivariate regression**, these findings could play a crucial role in developing more effective public health policies and interventions aimed at addressing the impact of media on adolescent eating behaviors.

### Limitations of the study

This study has several limitations that must be acknowledged. First, the cross-sectional design restricts the ability to establish causal relationships and the temporal sequencing of variables. As data were collected at a single point, it is challenging to determine whether Mukbang watching leads to increased hedonic hunger or if individuals with higher hedonic hunger are more inclined to watch Mukbang content. Additionally, the reliance on self-reported measures for Mukbang viewing habits and eating behaviors may introduce bias, as participants could underreport or overreport their consumption patterns, thereby affecting the accuracy of the findings. Furthermore, the study focuses exclusively on nursing students from Damanhour University in Egypt, which may limit the generalizability of the results to other populations or educational contexts. The exclusion of students with diagnosed eating or metabolic disorders may also overlook the impact of Mukbang watching on a broader range of individuals. Lastly, potential confounding variables, such as psychological conditions, cultural influences, and peer effects, were not thoroughly examined, which could have implications for the study’s conclusions.

## Conclusion

In conclusion, this study underscores the prevalence of Mukbang watching among university nursing students and its significant association with hedonic hunger. The findings indicate frequent exposure to Mukbang content may heighten cravings for pleasurable foods, potentially influencing unhealthy eating behaviors. Given the high levels of internet usage and the increasing popularity of food-related media among young adults, understanding these dynamics is essential for addressing potential health risks linked to poor dietary choices.

### Recommendations

Several recommendations can be made based on this study’s findings and limitations. First, future research should adopt longitudinal designs to explore causal relationships between Mukbang watching and hedonic hunger and examine the effects across different demographics and cultural contexts. Additionally, targeted interventions should be developed to educate nursing students about the potential impacts of Mukbang viewing on their eating behaviors and overall health, incorporating strategies that promote mindful eating and healthier food choices. It is also essential to provide psychological support services within nursing programs to assist students in managing stress and emotional eating, which may be exacerbated by media influences. Furthermore, educational institutions should consider integrating discussions about media literacy and its effects on eating behaviors into their curricula, empowering students to assess food-related media critically. Finally, advocating for policies that promote healthier food environments on campuses and within communities will be crucial in reducing the risk of unhealthy eating patterns driven by media consumption.

### Relevance to clinical practice

The findings of this study have significant implications for clinical practice, particularly in the fields of nutrition, nursing, and mental health. Understanding the relationship between Mukbang watching and hedonic hunger among nursing students can inform healthcare professionals about the potential risks associated with media consumption and its influence on dietary behaviors. As future healthcare providers, nursing students will play a crucial role in educating patients about healthy eating habits and the impact of lifestyle choices on overall health.

Clinicians can utilize these insights to develop targeted interventions to promote healthy eating behaviors among young adults. By recognizing the influence of food-related media, practitioners can create educational programs that emphasize mindful eating and critical evaluation of media content. This can help students and patients understand the potential for media to stimulate cravings and contribute to unhealthy eating patterns.

## Data Availability

The datasets used or analyzed in this study are available from the corresponding author upon request.
